# Effects of Transcranial Direct Current Stimulation on Knee Osteoarthritis Pain in Elderly Subjects With Defective Endogenous Pain-Inhibitory Systems: Protocol for a Randomized Controlled Trial

**DOI:** 10.2196/11660

**Published:** 2018-10-29

**Authors:** Daniela Regina Brandao Tavares, Jane Erika Frazao Okazaki, Aline Pereira Rocha, Marcia Valeria De Andrade Santana, Ana Carolina Pereira Nunes Pinto, Vinicius Tassoni Civile, Fania Cristina Santos, Felipe Fregni, Virginia Fernandes Moça Trevisani

**Affiliations:** 1 Department of Evidence-Based Medicine Brazilian Cochrane Centre Federal University of São Paulo Sao Paulo Brazil; 2 Department of Geriatrics and Gerontology Federal University of São Paulo Sao Paulo Brazil; 3 Department of Biological and Health Sciences Federal University of Amapá Amapá Brazil; 4 Institute of Health Sciences Paulista University Sao Paulo Brazil; 5 Laboratory of Neuromodulation & Center for Clinical Research Learning, Physics and Rehabilitation Department Spaulding Rehabilitation Hospital Harvard Medical School Boston, MA United States; 6 Department of Rheumatology Santo Amaro University Sao Paulo Brazil

**Keywords:** transcranial direct current stimulation, elderly, chronic pain, knee osteoarthritis, conditioned pain modulation

## Abstract

**Background:**

Knee osteoarthritis (OA) has been the main cause behind chronic pain and disabilities in the elderly population. The traditional treatment for knee OA pain currently concerns a number of combinations of pharmacological and nonpharmacological therapies. However, such combinations have displayed little effects on a significant group of subjects. In addition to this, pharmacological treatments often cause adverse effects, which limits their use on this population. Previous studies showed that chronic knee OA pain may be associated with maladaptive compensatory plasticity in pain-related neural central circuits indexed by a defective descending pain-inhibitory system. Transcranial direct current stimulation (tDCS) can revert some of these maladaptive changes, thus decreasing chronic pain sensation. Numerous studies have demonstrated that the use of anodal tDCS stimulation over the primary motor cortex (M1) has positive effects on chronic neuropathic pain. Yet, data on OA pain in elderly patients, including its effects on the endogenous pain-inhibitory system, remain limited.

**Objective:**

The objective of this study is to evaluate the efficacy of tDCS in reducing pain intensity caused by knee OA in elderly subjects with defective endogenous pain-inhibitory systems.

**Methods:**

We designed a randomized, sham-controlled, single-center, double-blinded clinical trial. Patients with knee OA who have maintained a chronic pain level during the previous 6 months and report a pain score of 4 or more on a 0-10 numeric rating scale (NRS) for pain in that period will undergo a conditioned pain modulation (CPM) task. Participants who present a reduced CPM response, defined as a decrease in NRS during the CPM task of less than 10%, and meet all of the inclusion criteria will be randomly assigned to receive 15 sessions of 2 mA active or sham tDCS for 20 minutes. A sample size of 94 subjects was calculated. The Brief Pain Inventory pain items will be used to assess pain intensity as our primary outcome. Secondary outcomes will include pain impact on functioning, mobility performance, quality of life, CPM, pressure pain threshold, touch-test sensory evaluation, and safety. Follow-up visits will be performed 2, 4, and 8 weeks following intervention. The data will be analyzed using the principle of intention-to-treat.

**Results:**

This study was approved by the institutional review board with the protocol number 1685/2016. The enrollment started in April 2018; at the time of publication of this protocol, 25 subjects have been enrolled. We estimate we will complete the enrollment process within 2 years.

**Conclusions:**

This clinical trial will provide relevant data to evaluate if anodal tDCS stimulation over M1 can decrease chronic knee OA pain in elderly subjects with defective CPM. In addition, this trial will advance the investigation of the role of central sensitization in knee OA and evaluate how tDCS stimulation may affect it.

**Trial Registration:**

ClinicalTrials.gov NCT03117231; https://clinicaltrials.gov/ct2/show/NCT03117231 (Archived by WebCite at http://webcitation.org/73WM1LCdJ)

**International Registered Report Identifier (IRRID):**

RR1-10.2196/11660

## Introduction

Osteoarthritis (OA) is the most common cause of pain and disabilities in elderly populations, and the knee is the most commonly affected joint [1,2]. OA is a disease characterized by cell stress and extracellular matrix degradation that stimulates maladaptive repair responses as proinflammatory pathways of innate immunity [3]. It has been determined that 33.6% of individuals older than 65 years suffer from OA, and 10% of individuals aged 60 years and older have symptomatic knee OA [4]. Subjects with chronic knee OA pain may experience depressive symptoms, sleep disorders, functional dependency, and a decrease in life quality, and this can have a subsequent impact on public health system costs [5]. OA knee pain is traditionally managed with a combination of pharmacological and nonpharmacological therapies including weight loss, physical therapy, several exercise modalities, anti-inflammatory drugs, topical agents, and opioids analgesics [6]. Although these treatments can decrease the pain intensity, they can also lead to substantial adverse effects (eg, dizziness, dry mouth, constipation, and nausea), and the treatment benefits may decrease over time (eg, opioid tolerance development) [7-9]. Previous studies suggested that peripheral and central sensitization are two of the underlying mechanisms in OA pain that can lead to lower pain thresholds, hyperalgesia, and spatial summation in asymptomatic areas [10,11].

Peripheral sensitization is caused by changes in the physiology of peripheral nociceptors due to localized inflammation. At the central level, there is an imbalance in endogenous pain modulation, characterized by a greater facilitation of pain and a reduced capacity to inhibit pain [12]. In addition, the aging process is associated with a dysregulation in the central modulation of pain, potentially placing elderly patients at a greater risk of chronic pain [13,14]. Conditioned pain modulation (CPM) is one of the most studied dynamic quantitative sensory tests; CPM is related to the reduction of pain produced by a test stimulus in response to a second noxious conditioning stimulus on a remote body side [15-17]. Previous studies showed that the pain reduction during CPM testing in healthy adults is approximately 23%, while in the healthy elderly population the reduction is approximately 17% [18,19]. A recent study indicated that elderly individuals with chronic pain due to severe osteoarthritis may have a significant dysfunction in the descending control of nociceptive processing, as the pain reduction during a CPM task is less than 10% [20].

In support of the above mentioned central sensitization mechanism, the rationale for treatment should also aim to modulate central nervous system plasticity [21,22]. Although drugs can change brain plasticity, their effects are more diffuse, which limits results. Moreover, as pharmacological treatments to chronic pain often lead to intolerable side effects in elderly patients, there is an increasing interest in nonpharmacological intervention options [23-25]. Transcranial direct current stimulation (tDCS) is a powerful noninvasive technique to modulate cortical excitability using a weak direct current applied through the scalp in a painless way; its prolonged and continuous application can induce neuroplasticity [26,27]. These changes depend on the polarity of the montage, with anodal stimulation enhancing excitability and cathodal stimulation decreasing excitability [28]. tDCS has shown positive effects on chronic pain in some diseases, which indicates that it may revert maladaptive plasticity associated with chronic pain sensation [29,30]. For pain, the most effective cortical target is the primary motor cortex (M1), a gateway to deep pain-related networks such as thalamic nuclei [31,32]. Previous brain imaging studies showed a positive cortical and subcortical neurophysiologic response to tDCS with anode over M1 and cathode over contralateral supraorbital region [33,34]. A recent trial showed that tDCS over the motor cortex might increase CPM response [35]. Another recent pilot trial evaluated the effect of tDCS on clinical pain severity for older adults with knee OA; it did not display consistent results in all pain assessments, yet their preliminary results showed promising clinical efficacy [36].

The aim of this trial is to assess the effects of tDCS on the intensity of pain in elderly patients suffering with OA knee chronic pain. We chose to test these effects in aged people with defective endogenous inhibitory pain systems, as the previous data showed increased therapeutic response in this population [37,38]. As secondary outcomes, we will evaluate the safety of tDCS and the changes in quality of life, pain impact on functioning, CPM, pressure pain threshold, touch-test sensory evaluation, and mobility performance.

## Methods

### Study Design

We will conduct a single-center, double-blinded, randomized, and controlled parallel clinical trial. The study was approved by the Ethics Committee of São Paulo Hospital (HSP) with the protocol number 1685/2016 and has been registered with ClinicalTrials.gov (NCT03117231). The trial will be performed at the Federal University of São Paulo, Brazil, and will be reported according to the Consolidated Standards of Reporting Trials (CONSORT) [39]. Participants will agree to the participation by signing the informed consent. They will be allowed to leave the study at any time without negative repercussions.

### Participants

Individuals aged 60 years and older, which is the definition for the elderly in Brazil [40], with an established diagnosis of unilateral or bilateral knee OA as established by the American College of Rheumatology criteria of 1986, using history, physical examination, and radiographic findings according to the Kellgren-Lawrence radiographic grading, will be recruited [41]. Patients will be recruited from HSP and from all the outpatient clinics belonging to the Federal University of São Paulo. Eligible subjects will have had pain in the knee for a minimum of 6 months, with an average pain score of 4 or more on a 0-10 numeric rating scale (NRS) for pain over that period and during the week prior to the first stimulation session. Following this, the subjects will be required to report a reduction on NRS during the CPM task of less than 10% compared to the pain score before the test.

Exclusion criteria include patients who have any severe acute or chronic uncompensated disease (such as uncontrolled hypertension, diabetes, cardiac issues, heart failure, or chronic obstructive pulmonary disease), history of epilepsy or syncope, contraindications to transcranial brain stimulation (ie, implanted brain medical devices or implanted brain metallic devices), established cognitive impairment, history of fractures in the lower limbs and/or spine in the last 6 months, traumatic brain injury with residual neurological deficits, alcohol abuse within the last 6 months as self-reported, use of carbamazepine within the last 6 months as self-reported, or severe depression (with a score of more than 30 on the Beck Depression Inventory). The principal investigator will obtain informed consent prior to enrolling participants in the study. We expect in this elderly population to have a female-to-male ratio of about 70%:30%.

In order to avoid drop outs, the principal investigator will follow up with all participants by phone during the entire study to remind them of the schedule. Participants and treating physicians will be told that changes in treatment during the study are strongly discouraged and must be reported. In addition, no treatment crossover will be permitted.

### Randomization and Blinding

We will use a computer-generated list to randomize subjects to one of the two treatment arms: active transcranial direct current stimulation (tDCS-a) or sham transcranial direct current stimulation (tDCS-s). The participants will be randomized with a ratio of 1:1 to either group using randomized block sizes (4 and 6) and stratified by severity of pain (less than 7 and 7 or more). The randomization will be concealed in consecutively numbered, sealed opaque envelopes and will be performed by an investigator not involved in the assessments or the recruitment. The participants and investigators who will perform the evaluation will be completely blinded until completion of the study and data processing.

### Intervention

The treatment will be administered for 3 weeks with intervention sessions daily from Monday to Friday for a total of 15 days of electrical stimulation [37]. The participants will not receive cash, reimbursements, food, transportation, or any compensation for their participation in the trial. After the initial assessment and before randomization, the subjects will be informed about the possible sensations with the stimulation and instructed not to reveal the sensation experienced.

Participants will be seated comfortably in an armchair while receiving tDCS. All subjects will receive 20 minutes of either sham or active tDCS, using a pair of 35 cm^2^ surface sponge electrodes soaked with physiologic saline and fixed to the head with elastic bands. The transcranial stimulation will be applied by a constant current device (Soterix 1×1 Low-Intensity Stimulator, Soterix Medical Inc) with an intensity of 2 mA. A gradual current ramp-up and ramp-down with 30-second durations will be used. The anode will be placed over C3/C4 (International 10-20 electroencephalogram system), which corresponds approximately to the location of M1 of the contralateral side to the most affected knee. The cathode electrode will be placed over the contralateral orbital, meaning ipsilateral to the most affected knee. The tDCS will be administered by a physician with specific training is this modality of intervention.

Sham tDCS stimulation will look and feel exactly the same as the active stimulation; however, the stimulator will deliver 2 mA of current for only 30 seconds. This sham stimulation method is frequently used and has been shown to be reliable [42].

**Figure 1:**
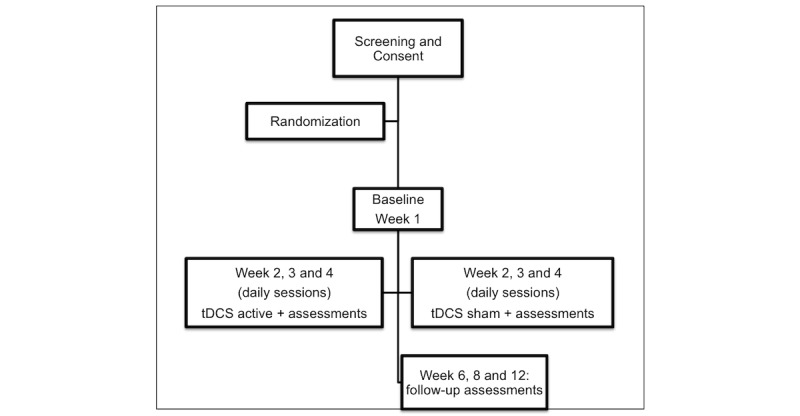
Flowchart of the study based on Consolidated Standards of Reporting Trials criteria. tDCS: transcranial direct current stimulation.

### Outcome Measurements

Following determination of participant eligibility and obtaining informed consent, we will collect baseline information about the subjects through a structured interview. Collected data will include sociodemographic information, mood and cognitive evaluation, radiographic data, and clinical history. We will perform all assessments during the baseline visit to obtain data to compare with the follow-up visits. Baseline data will be collected 1 week prior to the beginning of treatment. After 3 weeks of intervention, we will evaluate the participants over the course of 2 months, with 3 follow-up visits during this period (see Figure 1 for flow diagram). These outcome time points were chosen based on tDCS trials to assess early and late effects [27,36,37].

### Primary Outcome

We will analyze pain intensity in the previous 24 hours using the Brief Pain Inventory (BPI); this is a short, self-report questionnaire [43]. The BPI includes 4 items that measure pain intensity (pain right now, pain on average in last 24 hours, worst pain in last 24 hours, and least pain in last 24 hours) using an NRS from 0 (no pain) to 10 (pain as bad as you can imagine). The mean of these 4 pain items will be used as our primary outcome. The BPI also includes 7 items that assess the impact of the pain on functioning using a 0 (no interference) to 10 (complete interference) rating scale, which will be used as a secondary outcome.

### Secondary Outcomes

#### Quality of Life

We will assess health-related quality of life using the 12-Item Short Form Health Survey questionnaire, which comprises 8 domains that include physical functioning, physical role functioning, bodily pain, general health perceptions, vitality, social functioning, emotional role functioning, and mental health [44].

#### Pain Perception

Mechanical detection threshold (MDT) will be assessed using a Von Frey monofilament (VFM) as a mechanical stimulating device to evaluate light touch and pinprick sensation in small cutaneous regions. The investigator will use the smallest weighted monofilament, followed by sequentially larger monofilaments until the participant reports when the light touch sensation is first detected and then until the pinprick sensation is detected. VFM will be applied perpendicular to the skin with the correct amount of force to bow the monofilament for approximately 1.5 seconds. VFM will be applied first to the thenar region, ipsilateral to the most affected knee, and then applied to the most painful knee [45].

Mechanical pain threshold (MPT) will be measured using the same technique used to assess the MDT. However, the subject will be asked to report the smallest monofilament that produces pain. Following this, we will apply the same monofilament on either region 3 times, and the participants will be asked to rate the pain using an NRS; the mean score will then be calculated [45,46].

#### Pressure Pain Threshold

The pressure pain threshold (PPT) will be assessed using a pressure algometer (Commander Echo Algometer, JTECH Medical Industries Inc) that has a 1-cm^2^ rubber probe. The probe will be applied perpendicular to the skin until the subject first reports that the pressure sensation changes to a pain sensation. Participants will be asked to rate the pain using an NRS. The pressure will be applied to exactly the same regions that we will use to assess MDT and MPT [47].

#### Conditioned Pain Modulation

CPM will be measured using cold water as the conditioned stimulus and pressure as the test stimulus to assess endogenous pain modulation. Pressure will be applied using the same device and technique as for the PPT. Participants will be asked to immerse their contralateral hand to the most affected knee in a cold water bath maintained at 10^o^C for approximately 1 minute. In the last 30 seconds, the test stimulus will be applied following the PPT procedure [15-17].

We will calculate the percentage change in pain scores following CPM, a measurement we will call CPM-P, and the change in the PPT after the CPM that we will call CPM-PPT. Both of them will be calculated using the following formula: (post-CPM trial score − pre-CPM trial score / pre-CPM trial score) × 100. For CPM-P, a negative score will indicate pain inhibition after the conditioning stimulus. For CPM-PPT, a positive score will indicate pain inhibition after the conditioning stimulus.

#### Physical Functioning

The Western Ontario and McMaster Universities Osteoarthritis Index is a valid and reliable instrument commonly used to assess pain and disability in studies of knee OA. The questionnaire includes 3 subscales to assess pain, perceived disability, and joint stiffness. The total score ranges from 0 to 96, with higher values indicating greater physical impairment [48].

The Lequesne Algofunctional Index is a questionnaire that assesses 3 components of severity of OA: pain, maximum walking distance, and activities of daily living. The total score ranges from 0 to 24, with higher values indicating greater physical impairment [49].

The timed up and go test is a reliable and practical test to evaluate functional capacity commonly used in research studying elderly populations, especially because of its capacity to assess risk of falls and gait function. Subjects will be asked to get up from a chair in which they were fully supported, walk 3 meters, turn around, return by the same route, and sit back down on the chair with their back supported. We will measure the time required to complete the test. The test will be performed twice, and the lowest score will be recorded [50-52].

The one leg stance test is used to evaluate postural balance. The participants will be asked to stand unsupported on one foot while looking straight ahead with their hands on hips. We will measure the time the subject is able to maintain balance until the contralateral foot touches the ground. The test will be performed twice, and the highest score will be recorded [52,53].

#### Pain Impact on Functioning

The mean of the 7 BPI interference items (general activity, mood, walking ability, normal work, relations with other people, sleep, enjoyment of life) will be used to assess the pain impact on functioning.

#### Patient Global Assessment

We will analyze the patient’s global assessment on how the intervention affects the patient’s status using a 0-100 visual analog scale (VAS) scale [54].

#### Adverse Effects

At each stimulation session and at all follow-ups, participants will be administered a questionnaire to evaluate potential adverse effects of stimulation on a 5-point scale. The main potential adverse effects include headache, neck and scalp pain, tingling, sleepiness, and acute mood change. If any side effect is reported, its potential relationship with the intervention will be assessed [55].

To assess the safety of tDCS, we will analyze the mood and cognition of the subjects at each stimulation session and at all follow-ups. Mood will be assessed with a 0-100 VAS scale by asking the subject to rate their own emotions including anxiety, depression, stress, and sleepiness. Cognition will be analyzed using the Mini-Mental State Examination, a brief screening of cognitive abilities [56].

### Sample Size and Data Analysis

The sample size was estimated using the minimal clinically important difference (20% reduction from the baseline) in the outcome of pain intensity measured by the NRS as a parameter. The effect size and the probability of error type I (alpha) and type II (beta) were 0.59, .05, and .2, respectively. According to the sample calculation, that total size would be 94 participants. We will increase the sample size by 10% to account for dropouts and other unexpected factors. Thus, the final sample size is 104 subjects.

All analyses will be conducted according to the principle of intention-to-treat (ITT) using a regression-based single imputation method. The ITT population will include all randomized subjects. We will perform an additional sensitivity analysis in which we will use the method of multiple imputation. Initially, the baseline characteristics between sham and tDCS groups will be tested using Student *t* tests; to analyze our primary outcome, which is pain intensity measured by BPI, we will adopt a mixed analysis of variance model in which the dependent variable will be the outcome (BPI) and the independent variables will be group (tDCS-a or tDCS-s), time (baseline and after treatment and follow-up), and the interaction group time. Secondary analyses will be conducted in an exploratory manner (no correction for multiple comparisons). Similar analysis will be conducted for the adverse effects measuring continuous outcomes, and for the categorical outcomes, we will use Fisher exact tests. In both cases, for the safety analysis, we will use uncorrected *P* values to increase the likelihood of detecting detrimental adverse effects.

## Results

This clinical trial began enrollment in April 2018. As of publication, 40 subjects have been evaluated, 25 have been included satisfactorily, and 10 participants have completed the entire protocol. We estimate enrollment will be completed within 2 years. Results will be presented at scientific meetings and published in peer-reviewed journals. All publications will be authorized by the study investigators.

## Discussion

This study will assess a relevant public health problem, as the overall population is getting older and the prevalence of chronic disorders is increasing. This randomized clinical trial will evaluate the effects of the cumulative noninvasive brain stimulation technique as an instrument to decrease pain intensity by inducing neuroplasticity in brain circuits related to central sensitization. As a secondary effect, we will observe impact on the quality of life and mobility performance in elderly patients with knee OA.

This trial will contribute to understanding the underlying mechanisms of the analgesic effects of tDCS over the motor cortex by assessing the CPM response of the subjects. In this trial, we are only enrolling subjects with defective endogenous inhibitory pain system. We chose this criteria given the mechanisms of tDCS, as well as effects of tDCS on this system [57-58]. Therefore, this study will be important to preliminarily test the suitability of CPM as a valuable prognostic and surrogate marker. Finding better neurophysiological markers for the use of tDCS is critical to advance this field. A potential significant correlation between pain scores and CPM response will indicate that CPM may be a useful marker.

Most interventions for OA do not target the central sensitization and defective descending inhibitory pain system. For instance, nonsteroidal anti-inflammatory drugs inhibit the two recognized forms of prostaglandin synthase, thus providing an analgesic and anti-inflammatory effect; however, their prolonged use is limited because they carry risk for cardiovascular and upper gastrointestinal diseases [59,60]. Thus, tDCS can be important for nonresponders to the traditional treatment. This intervention has the opposite effect on the endogenous pain system when compared to opioids, which, when used chronically, may increase sensitivity to a noxious stimulus and consequently induce hyperalgesia [61].

Another important finding from this trial concerns the additional data we will provide on the tDCS effects in elderly populations. While there have been a fair number of trials testing tDCS in chronic pain, there have been few randomized clinical trials testing tDCS for chronic pain in elderly patients. To date, there have been no well-powered trials (such as this one with 104 subjects) designed to test a homogenous population [29,62,63]. We believe that this trial can provide important information to advance this therapy in this population.

We decided to use 15 stimulation sessions based on a recent trial on chronic pain due to fibromyalgia, which estimated 15 tDCS sessions as the median number of stimulations to produce clinically meaningful outcomes [37].

The safety of tDCS has been well established, and after several studies, researchers concluded that tDCS induces only temporary mild effects. The most common side effects include headache, dizziness, nausea, mild pain, itchy sensation, and skin redness under area of the electrodes [26,38,55-57]. However, it is important to assess safety in elderly patients given that there is much less experience with this population. This trial will provide additional data for this domain, which will be useful when planning future trials.

Some concerns about the study should be discussed. One of the possible issues may be the combination with other therapies, which may influence the results. Given the relatively large sample size of subjects, we believe that this potential covariate will be balanced in the two groups. In addition, subjects will be asked to remain stable in their drug treatment, and any changes will be noted for secondary subgroup analysis. Finally, although blinding is not perfect in tDCS and 10% to 20% may become unblinded, we have demonstrated in longitudinal trials that there are no blinding-related biases in a tDCS clinical trial for a parallel design [64]. This trial has several strengths, such as a relatively large sample (one of the largest in tDCS pain research) and a population selected based on the endogenous mechanisms of tDCS (CPM).

## Abbreviations

BPI: Brief Pain Inventory
CPM: conditioned pain modulation
CPM-P: percentage change in pain score following conditioned pain modulation
CPM-PPT: change in pressure pain threshold after conditioned pain modulation
CONSORT: Consolidated Standards of Reporting Trials
HSP: Sao Paulo Hospital
ITT: intention-to-treat
M1: primary motor cortex
MDT: mechanical detection threshold
MPT: mechanical pain threshold
NRS: numeric rating scale
OA: osteoarthritis
PPT: pressure pain threshold
tDCS: transcranial direct current stimulation
tDCS-a: active transcranial direct current stimulation
tDCS-s: sham transcranial direct current stimulation
VAS: visual analog scale
VFM: Von Frey monofilament

## References

[1] Heidari B (2011). Knee osteoarthritis prevalence, risk factors, pathogenesis and features: part I. Caspian J Intern Med.

[2] Lawrence RC, Felson DT, Helmick CG, Arnold LM, Choi H, Deyo RA, Gabriel S, Hirsch R, Hochberg MC, Hunder GG, Jordan JM, Katz JN, Kremers HM, Wolfe F (2008). Estimates of the prevalence of arthritis and other rheumatic conditions in the United States. Part II. Arthritis Rheum.

[3] Hunter DJ, McDougall JJ, Keefe FJ (2008). The symptoms of osteoarthritis and the genesis of pain. Rheum Dis Clin North Am.

[4] Woolf AD, Pfleger B (2003). Burden of major musculoskeletal conditions. Bull World Health Organ.

[5] Benazzo F, Perticarini L, Padolino A, Castelli A, Gifuni P, Lovato M, Manzini C, Giordan N (2016). A multi-centre, open label, long-term follow-up study to evaluate the benefits of a new viscoelastic hydrogel (Hymovis) in the treatment of knee osteoarthritis. Eur Rev Med Pharmacol Sci.

[6] Hochberg MC, Altman RD, April KT, Benkhalti M, Guyatt G, McGowan J, Towheed T, Welch V, Wells G, Tugwell P (2012). American College of Rheumatology 2012 recommendations for the use of nonpharmacologic and pharmacologic therapies in osteoarthritis of the hand, hip, and knee. Arthritis Care Res (Hoboken).

[7] Chaparro LE, Furlan AD, Deshpande A, Mailis-Gagnon A, Atlas S, Turk DC (2014). Opioids compared with placebo or other treatments for chronic low back pain: an update of the Cochrane Review. Spine (Phila Pa 1976).

[8] Ho KY, Chua NH, George JM, Yeo SN, Main NB, Choo CY, Tan JW, Tan KH, Ng BY, Pain Association of Singapore Task Force (2013). Evidence-based guidelines on the use of opioids in chronic non-cancer pain—a consensus statement by the Pain Association of Singapore Task Force. Ann Acad Med Singapore.

[9] Reinecke H, Weber C, Lange K, Simon M, Stein C, Sorgatz H (2015). Analgesic efficacy of opioids in chronic pain: recent meta-analyses. Br J Pharmacol.

[10] Lluch E, Torres R, Nijs J, Van Oosterwijck J (2014). Evidence for central sensitization in patients with osteoarthritis pain: a systematic literature review. Eur J Pain.

[11] Arendt-Nielsen L, Nie H, Laursen MB, Laursen BS, Madeleine P, Simonsen OH, Graven-Nielsen T (2010). Sensitization in patients with painful knee osteoarthritis. Pain.

[12] Imamura M, Imamura ST, Kaziyama HHS, Targino RA, Hsing WT, de Souza LP, Cutait MM, Fregni F, Camanho GL (2008). Impact of nervous system hyperalgesia on pain, disability, and quality of life in patients with knee osteoarthritis: a controlled analysis. Arthritis Rheum.

[13] Edwards RR, Fillingim RB (2001). Effects of age on temporal summation and habituation of thermal pain: clinical relevance in healthy older and younger adults. J Pain.

[14] Edwards RR, Fillingim RB, Ness TJ (2003). Age-related differences in endogenous pain modulation: a comparison of diffuse noxious inhibitory controls in healthy older and younger adults. Pain.

[15] Lewis GN, Heales L, Rice DA, Rome K, McNair PJ (2012). Reliability of the conditioned pain modulation paradigm to assess endogenous inhibitory pain pathways. Pain Res Manag.

[16] Lewis GN, Rice DA, McNair PJ (2012). Conditioned pain modulation in populations with chronic pain: a systematic review and meta-analysis. J Pain.

[17] Kennedy DL, Kemp HI, Ridout D, Yarnitsky D, Rice ASC (2016). Reliability of conditioned pain modulation: a systematic review. Pain.

[18] Moont R, Pud D, Sprecher E, Sharvit G, Yarnitsky D (2010). "Pain inhibits pain" mechanisms: is pain modulation simply due to distraction?. Pain.

[19] Naugle KM, Ohlman T, Naugle KE, Riley ZA, Keith NR (2017). Physical activity behavior predicts endogenous pain modulation in older adults. Pain.

[20] Tarragó Mda G, Deitos A, Brietzke AP, Vercelino R, Torres IL, Fregni F, Caumo W (2016). Descending control of nociceptive processing in knee osteoarthritis is associated with intracortical disinhibition: an exploratory study. Medicine (Baltimore).

[21] Fregni F, Pascual-Leone A, Freedman SD (2007). Pain in chronic pancreatitis: a salutogenic mechanism or a maladaptive brain response?. Pancreatology.

[22] Zimmermann M (1989). Pain mechanisms and mediators in osteoarthritis. Semin Arthritis Rheum.

[23] O'Neil CK, Hanlon JT, Marcum ZA (2012). Adverse effects of analgesics commonly used by older adults with osteoarthritis: focus on non-opioid and opioid analgesics. Am J Geriatr Pharmacother.

[24] Solomon DH, Rassen JA, Glynn RJ, Lee J, Levin R, Schneeweiss S (2010). The comparative safety of analgesics in older adults with arthritis. Arch Intern Med.

[25] Reid MC, Henderson CR, Papaleontiou M, Amanfo L, Olkhovskaya Y, Moore AA, Parikh SS, Turner BJ (2010). Characteristics of older adults receiving opioids in primary care: treatment duration and outcomes. Pain Med.

[26] Nitsche MA, Cohen LG, Wassermann EM, Priori A, Lang N, Antal A, Paulus W, Hummel F, Boggio PS, Fregni F, Pascual-Leone A (2008). Transcranial direct current stimulation: state of the art 2008. Brain Stimul.

[27] Fregni F, Nitsche MA, Loo CK, Brunoni AR, Marangolo P, Leite J, Carvalho S, Bolognini N, Caumo W, Paik NJ, Simis M, Ueda K, Ekhitari H, Luu P, Tucker DM, Tyler WJ, Brunelin J, Datta A, Juan CH, Venkatasubramanian G, Boggio PS, Bikson M (2015). Regulatory considerations for the clinical and research use of transcranial direct current stimulation (tdcs): review and recommendations from an expert panel. Clin Res Regul Aff.

[28] Nitsche MA, Paulus W (2000). Excitability changes induced in the human motor cortex by weak transcranial direct current stimulation. J Physiol.

[29] Zaghi S, Heine N, Fregni F (2009). Brain stimulation for the treatment of pain: a review of costs, clinical effects, and mechanisms of treatment for three different central neuromodulatory approaches. J Pain Manag.

[30] Fregni F, Freedman S, Pascual-Leone A (2007). Recent advances in the treatment of chronic pain with non-invasive brain stimulation techniques. Lancet Neurol.

[31] Jensen MP, Day MA, Miró J (2014). Neuromodulatory treatments for chronic pain: efficacy and mechanisms. Nat Rev Neurol.

[32] Jensen MP, Hakimian S, Sherlin LH, Fregni F (2008). New insights into neuromodulatory approaches for the treatment of pain. J Pain.

[33] Polanía R, Paulus W, Nitsche MA (2012). Modulating cortico-striatal and thalamo-cortical functional connectivity with transcranial direct current stimulation. Hum Brain Mapp.

[34] Antal A, Polania R, Schmidt-Samoa C, Dechent P, Paulus W (2011). Transcranial direct current stimulation over the primary motor cortex during fMRI. Neuroimage.

[35] Flood A, Waddington G, Cathcart S (2016). High-definition transcranial direct current stimulation enhances conditioned pain modulation in healthy volunteers: a randomized trial. J Pain.

[36] Ahn H, Woods AJ, Kunik ME, Bhattacharjee A, Chen Z, Choi E, Fillingim RB (2017). Efficacy of transcranial direct current stimulation over primary motor cortex (anode) and contralateral supraorbital area (cathode) on clinical pain severity and mobility performance in persons with knee osteoarthritis: an experimenter- and participant-blinded, randomized, sham-controlled pilot clinical study. Brain Stimul.

[37] Castillo-Saavedra L, Gebodh N, Bikson M, Diaz-Cruz C, Brandao R, Coutinho L, Truong D, Datta A, Shani-Hershkovich R, Weiss M, Laufer I, Reches A, Peremen Z, Geva A, Parra LC, Fregni F (2016). Clinically effective treatment of fibromyalgia pain with high-definition transcranial direct current stimulation: phase ii open-label dose optimization. J Pain.

[38] Reidler JS, Mendonca ME, Santana MB, Wang X, Lenkinski R, Motta AF, Marchand S, Latif L, Fregni F (2012). Effects of motor cortex modulation and descending inhibitory systems on pain thresholds in healthy subjects. J Pain.

[39] Gray R, Sullivan M, Altman DG, Gordon-Weeks AN (2012). Adherence of trials of operative intervention to the CONSORT statement extension for non-pharmacological treatments: a comparative before and after study. Ann R Coll Surg Engl.

[40] Paiva M, Ferrer N, Villarouco V (2015). The process of aging: a case study approach implementing an ergonomics evaluation of the built environment for the elderly in Brazil. Work.

[41] Altman R, Asch E, Bloch D, Bole G, Borenstein D, Brandt K, Christy W, Cooke TD, Greenwald R, Hochberg M (1986). Development of criteria for the classification and reporting of osteoarthritis. Classification of osteoarthritis of the knee. Diagnostic and Therapeutic Criteria Committee of the American Rheumatism Association. Arthritis Rheum.

[42] Gandiga PC, Hummel FC, Cohen LG (2006). Transcranial DC stimulation (tDCS): a tool for double-blind sham-controlled clinical studies in brain stimulation. Clin Neurophysiol.

[43] Kapstad H, Hanestad BR, Langeland N, Rustøen T, Stavem K (2008). Cutpoints for mild, moderate and severe pain in patients with osteoarthritis of the hip or knee ready for joint replacement surgery. BMC Musculoskelet Disord.

[44] Hungerer S, Kiechle M, von Ruden C, Militz M, Beitzel K, Morgenstern M (2017). Knee arthrodesis versus above-the-knee amputation after septic failure of revision total knee arthroplasty: comparison of functional outcome and complication rates. BMC Musculoskelet Disord.

[45] Walk D, Sehgal N, Moeller-Bertram T, Edwards RR, Wasan A, Wallace M, Irving G, Argoff C, Backonja M (2009). Quantitative sensory testing and mapping: a review of nonautomated quantitative methods for examination of the patient with neuropathic pain. Clin J Pain.

[46] Keizer D, van Wijhe M, Post WJ, Wierda JM (2007). Quantifying allodynia in patients suffering from unilateral neuropathic pain using Von Frey monofilaments. Clin J Pain.

[47] Kinser AM, Sands WA, Stone MH (2009). Reliability and validity of a pressure algometer. J Strength Cond Res.

[48] Bellamy N, Buchanan WW, Goldsmith CH, Campbell J, Stitt LW (1988). Validation study of WOMAC: a health status instrument for measuring clinically important patient relevant outcomes to antirheumatic drug therapy in patients with osteoarthritis of the hip or knee. J Rheumatol.

[49] Faucher M, Poiraudeau S, Lefevre-Colau MM, Rannou F, Fermanian J, Revel M (2003). Assessment of the test-retest reliability and construct validity of a modified Lequesne index in knee osteoarthritis. Joint Bone Spine.

[50] Steffen TM, Hacker TA, Mollinger L (2002). Age- and gender-related test performance in community-dwelling elderly people: Six-Minute Walk Test, Berg Balance Scale, Timed Up & Go Test, and gait speeds. Phys Ther.

[51] Shumway-Cook A, Brauer S, Woollacott M (2000). Predicting the probability for falls in community-dwelling older adults using the Timed Up & Go Test. Phys Ther.

[52] Lee K, Lee YW (2017). Efficacy of ankle control balance training on postural balance and gait ability in community-dwelling older adults: a single-blinded, randomized clinical trial. J Phys Ther Sci.

[53] Jonsson E, Seiger A, Hirschfeld H (2004). One-leg stance in healthy young and elderly adults: a measure of postural steadiness?. Clin Biomech (Bristol, Avon).

[54] Bellamy N, Kirwan J, Boers M, Brooks P, Strand V, Tugwell P, Altman R, Brandt K, Dougados M, Lequesne M (1997). Recommendations for a core set of outcome measures for future phase III clinical trials in knee, hip, and hand osteoarthritis. Consensus development at OMERACT III. J Rheumatol.

[55] Brunoni AR, Amadera J, Berbel B, Volz MS, Rizzerio BG, Fregni F (2011). A systematic review on reporting and assessment of adverse effects associated with transcranial direct current stimulation. Int J Neuropsychopharmacol.

[56] Iyer MB, Mattu U, Grafman J, Lomarev M, Sato S, Wassermann EM (2005). Safety and cognitive effect of frontal DC brain polarization in healthy individuals. Neurology.

[57] Hamner JW, Villamar MF, Fregni F, Taylor JA (2015). Transcranial direct current stimulation (tDCS) and the cardiovascular responses to acute pain in humans. Clin Neurophysiol.

[58] Woods AJ, Antal A, Bikson M, Boggio PS, Brunoni AR, Celnik P, Cohen LG, Fregni F, Herrmann CS, Kappenman ES, Knotkova H, Liebetanz D, Miniussi C, Miranda PC, Paulus W, Priori A, Reato D, Stagg C, Wenderoth N, Nitsche MA (2016). A technical guide to tDCS, and related non-invasive brain stimulation tools. Clin Neurophysiol.

[59] Bhala N, Emberson J, Merhi A, Abramson S, Arber N, Baron JA, Bombardier C, Cannon C, Farkouh ME, FitzGerald GA, Goss P, Halls H, Hawk E, Hawkey C, Hennekens C, Hochberg M, Holland LE, Kearney PM, Laine L, Lanas A, Lance P, Laupacis A, Oates J, Patrono C, Schnitzer TJ, Solomon S, Tugwell P, Wilson K, Wittes J, Baigent C, Coxibtraditional NSAID Trialists' (CNT) Collaboration (2013). Vascular and upper gastrointestinal effects of non-steroidal anti-inflammatory drugs: meta-analyses of individual participant data from randomised trials. Lancet.

[60] Bruyère O, Cooper C, Pelletier J, Branco J, Luisa BM, Guillemin F, Hochberg MC, Kanis JA, Kvien TK, Martel-Pelletier J, Rizzoli R, Silverman S, Reginster J (2014). An algorithm recommendation for the management of knee osteoarthritis in Europe and internationally: a report from a task force of the European Society for Clinical and Economic Aspects of Osteoporosis and Osteoarthritis (ESCEO). Semin Arthritis Rheum.

[61] Zhang Y, Ahmed S, Vo T, St Hilaire K, Houghton M, Cohen AS, Mao J, Chen L (2015). Increased pain sensitivity in chronic pain subjects on opioid therapy: a cross-sectional study using quantitative sensory testing. Pain Med.

[62] Harvey M, Lorrain D, Martel M, Bergeron-Vezina K, Houde F, Séguin M, Léonard G (2017). Can we improve pain and sleep in elderly individuals with transcranial direct current stimulation? Results from a randomized controlled pilot study. Clin Interv Aging.

[63] Concerto C, Al Sawah M, Chusid E, Trepal M, Taylor G, Aguglia E, Battaglia F (2016). Anodal transcranial direct current stimulation for chronic pain in the elderly: a pilot study. Aging Clin Exp Res.

[64] Brunoni AR, Schestatsky P, Lotufo PA, Benseñor IM, Fregni F (2014). Comparison of blinding effectiveness between sham tDCS and placebo sertraline in a 6-week major depression randomized clinical trial. Clin Neurophysiol.

